# Utility of the gastro-laryngeal tube during transesophageal echocardiography: A prospective randomized clinical trial

**DOI:** 10.1097/MD.0000000000032269

**Published:** 2022-12-16

**Authors:** Muhittin Calim, Harun Uysal, Nuray Kahraman Ay, Kazim Karaaslan, Hayrettin Daskaya

**Affiliations:** a Department of Anesthesiology and Reanimation, Bezmialem Vakif University School of Medicine, Istanbul, Turkey; b Department of Cardiology, Bezmialem Vakif University School of Medicine, Istanbul, Turkey.

**Keywords:** airway management, gastro-laryngeal tube, satisfaction, sedation and analgesia, transesophageal echocardiography

## Abstract

To validate the utility and performance of the gastro-laryngeal tube (GLT) in terms of cardiologist and patient satisfaction levels, incidence of and attempts at successful transesophageal echocardiography (TEE) probe placement, perioperative and postoperative hemodynamics, and adverse events related to the TEE procedure.

**Methods:**

In this randomized prospective clinical study, forty-four patients undergoing TEE and aged 20 to 80 years old scheduled for TEE were randomly allocated to two study groups: Group SA (sedation and analgesia) and Group GLT. Cardiologist and patient satisfaction levels, TEE probe placement performance, hemodynamics, adverse events related to the TEE procedure, demographic characteristics, and TEE procedure data were recorded.

**Results:**

The cardiologist satisfaction level was significantly higher in Group GLT (*P* = .011). The TEE probe was successfully placed at the first attempt in all the patients in Group GLT and at the first attempt in 11 patients, at the second attempt in 8 patients, and at the third attempt in 3 patients in Group SA. The TEE probe placement success was significantly higher in Group GLT (*P* < .001), and TEE probe placement was significantly easier in Group GLT (*P* < .001). There were no significant differences in patient satisfaction, heart rate, mean arterial pressure, oxygen saturation, adverse events related to the TEE procedure between the groups.

**Conclusion:**

The present study revealed that GLT use elicited a higher cardiologist satisfaction level and resulted in more successful and easier TEE probe placement. We thus conclude that the use of the recently developed GLT may ensure airway management safety and a comfortable TEE experience.

## 1. Introduction

Transesophageal echocardiography (TEE) is an ultrasonic viewing method that provides anatomic, functional, and hemodynamic information by viewing the heart and thoracic vascular structures using a probe advanced through the esophagus toward the stomach. The probe, with an ultrasonic wave converter at the tip, is placed in the patient’s esophagus and begins assessment at the closest location to the heart.^[1]^ This position allows for a detailed assessment of the heart. It is commonly used during heart surgery but is also used for diagnostic purposes in outpatient cardiology examinations.^[2]^

It is considered that TEE views are superior to transthoracic echocardiography.^[3]^ The presence of the TEE probe in the oropharyngeal passage and the need for deep sedation and analgesia in TEE pose high complication risks.^[4]^ That is, during sedation and analgesia, the common use of the oropharyngeal passage by both the cardiologist and anesthesiologist poses an airway management safety risk. Thus, especially during deep sedation and analgesia, special equipment and manipulation may be required to ensure airway management safety.^[5,6]^

**Figure 1.**
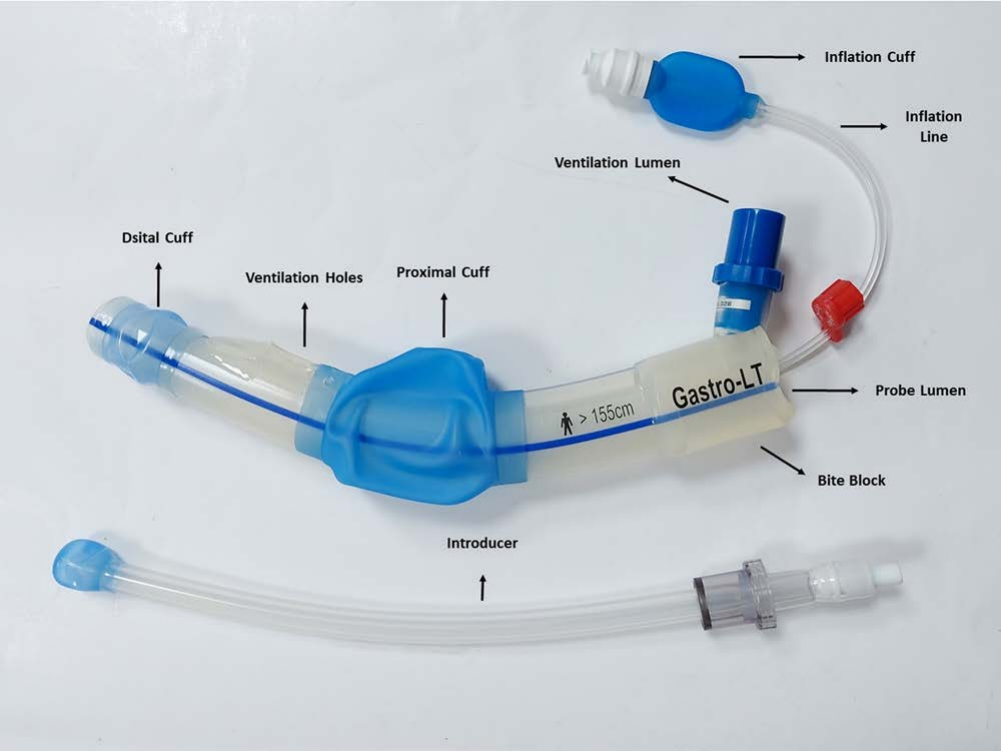
Gastro-laryngeal tube.

**Figure 2.**
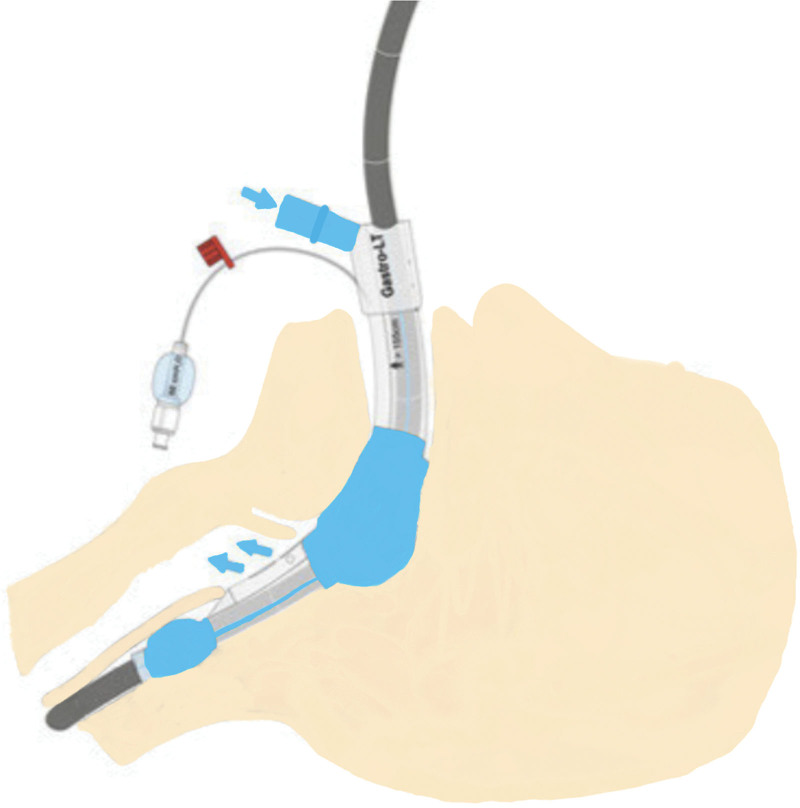
Anatomic placement of gastro-laryngeal tube.

The importance given to patient comfort and satisfaction and endoscopists’ desire to perform procedures more reliably and with shared responsibility have opened the door to new research and innovations.^[7]^ These demands have motivated the industry to develop new pharmaceutical agents and medical instruments. The gastro-laryngeal tube (GLT) was designed to ensure airway management safety and the optimum performance of endoscopy for esophageal procedures under deep sedation, and is a new tool offered for use (Fig. 1). GLT is a modification of the laryngeal tube, which allows the passage of the gastroscope or TEE probe. It is a suitable channel for manipulation and ensuring supraglottic airway control at the same time.^[8]^ It has two inflatable cuffs, one within the esophagus and the other in the hypopharynx. These cuffs ensure airway safety and GLT stabilization (Fig. 2).

We hypothesized that GLT, allowing passage of the TEE probe and ensuring supraglottic airway control, would provide more advantages in terms of hemodynamic stabilization, airway management safety, and cardiologist and patient satisfaction levels when used in a clinical setting. Prior to the present study, no detailed prospective clinical study had analyzed the performance of GLT during TEE. We conducted the present study to address such gap. The primary outcome measure was cardiologist satisfaction levels, and the secondary outcome measures were incidence of and attempts at successful TEE probe placement, patient satisfaction, demographic characteristics, procedure data, perioperative and postoperative hemodynamics, and adverse events related to the TEE procedure.

## 2. Methods

### 2.1. Study protocol

The present study was approved by Bezmialem Vakif University Clinical Research Ethics Committee (Decision no: 11/8, date: 03.06.2015) and registered with the U.S. National Institutes of Health (ClinicalTrials.gov; #NCT05272306). Informed consent was obtained from the patients for publication of this clinical trial details. We carried on a prospective clinical study with 44 patients underwent TEE within the period from April 13 to September 20, 2022 at a university hospital. It was prepared with respect to the Consolidated Standards of Reporting Trials.^[9]^

### 2.2. Study participants

The study participants (n = 44) were patients undergoing TEE with American Society of Anesthesiology (ASA) physical status I–III and aged 20 to 80 years old. We hold a meeting with all the patients during the study period before the TEE procedure to get their written informed consent to get involved in the study. In our hospital, the TEE procedure is routinely performed with sedation/analgesia in two days a week for the last three years. Patients who were under 20 or over 80 years old, were allergic to anesthetic drugs, had undergone an emergent procedure, had an uncontrolled cerebrovascular disease, had drug and alcohol addiction, had undergone oropharyngeal surgery, or refused to provide informed consent to get involved in the study were excluded from the study.

### 2.3. Preoperative procedures

Preoperative evaluations were applied on all the patients a day before the TEE procedure. Each patient’s age, gender, height, weight, and ASA physical status were recorded. On the day of the procedure, the patients were taken to the procedure room after premedication with 0.003 mg/kg midazolam administered via an intravenous (IV) route. Heart rate (HR), mean arterial pressure (MAP), electrocardiogram, and the peripheral oxygen saturation (SpO_2_) through pulse oximetry were used as a standard monitoring procedures.

### 2.4. Study design

**Figure 3.**
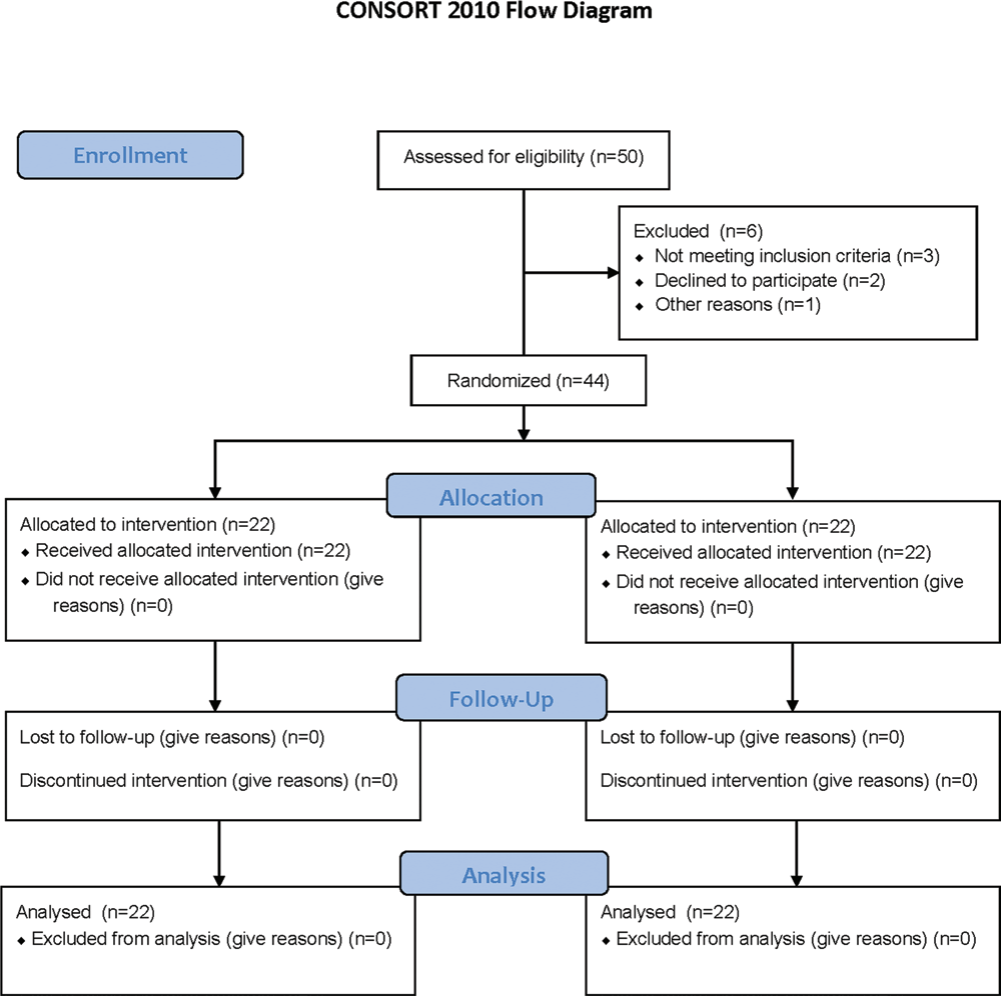
Flow diagram.

The present study was designed as a randomized prospective clinical study. As shown in Figure 3, 50 patients were randomly allocated to either Group SA (n = 25; sedation and analgesia) or Group GLT (n = 25; airway control provided by GLT). The randomization was based on a random number table generated by the MedCalc v.16 statistical software for Windows (medcalc.com.tr). After the allocation, 3 patients who did not meet the inclusion criteria, 2 patients declining to participate and a patient being canceled the procedure were excluded from the study. We ultimately included a total of 44 patients (Group SA; n = 22, Group GLT; n = 22) after dropout. All the patients received the standard intervention procedures decided by the same team of cardiologists and an anesthesiologist experienced in TEE and insertion of GLT. The standard intervention procedure protocol was not changed in any way. Before patient recruitment, the responsible anesthesiologist conducted a training session with all the anesthesia collaborators.

### 2.5. Anesthesia procedure

All the patients received preoperative midazolam before being taken to the procedure room. For the patients in Group SA, standardized sedation and analgesia were administered by an experienced anesthesiologist. After administration of lidocaine (1 mg/kg) and atropine sulfate (0.01 mg/kg) and after preoxygenation (100% 4 L/min O_2_ for 3 min), anesthesia was induced with propofol (1–2 mg/kg) and fentanyl (1–2 mcg/kg). The anesthesia was maintained with propofol infusion (4 mg/kg/h). Repetitive IV boluses of propofol (0.1 mg/kg) were administered if required. The patients protecting spontaneously breathing received oxygen (100%, 3 L/min) through a nasal cannula during sedation and analgesia.

**Figure 4.**
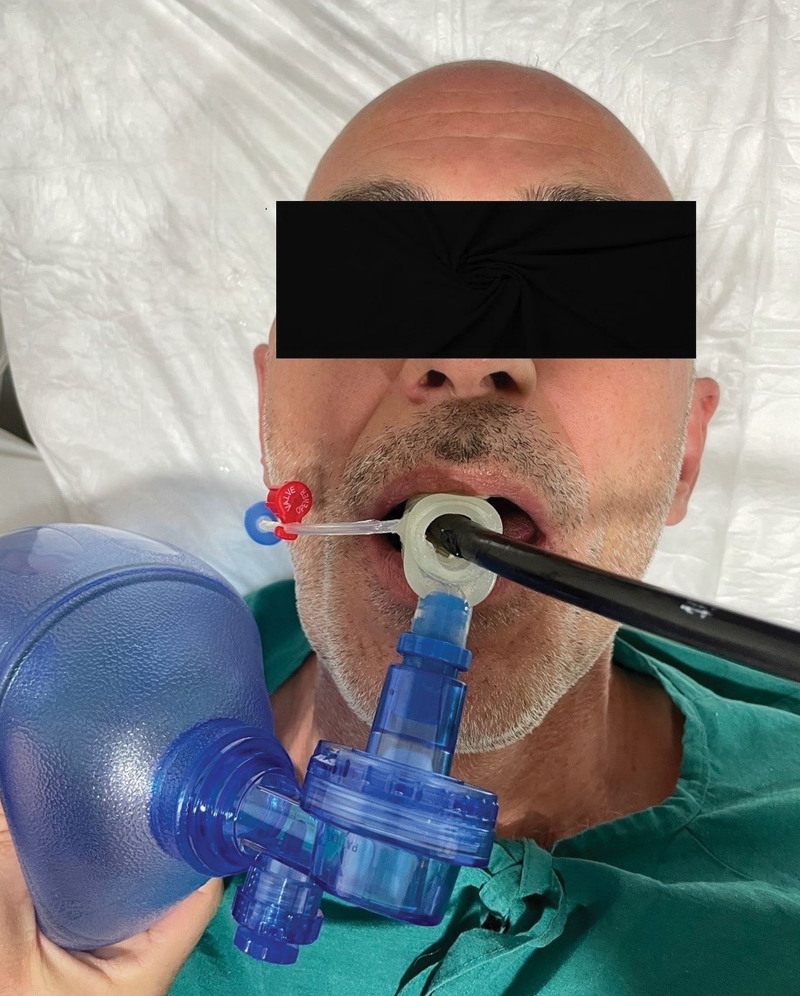
Gastro-laryngeal tube use during the procedure.

For the patients in Group GLT, after administration of lidocaine (1 mg/kg) and atropine sulfate (0.01 mg/kg) and after preoxygenation (100% 4 L/min O_2_ for 3 min), anesthesia was induced with propofol (1–2 mg/kg) and fentanyl (1–2 mcg/kg). Thereafter, a GLT (VBM Medizintechnik GmbH, Sulz, Germany) was inserted (Fig. 4). The patients were then ventilated manually with appropriate tidal volume (6–8 mL/kg) and frequency (12–14 breaths/min) using a bag-valve-mask ventilation system. Repetitive IV boluses of propofol (0.1 mg/kg) were administered if required. And also, we did not use inhalation anesthetics any time during the procedures. The GLT was removed at the end of the TEE procedure.

We applied to all patients standard monitoring during the entire procedure. The anesthesiologist was responsible for patient comfort, hemodynamic stability, immobility, adequate analgesia, and airway management.

### 2.6. Postoperative management

After the TEE procedure, the patients were transferred to the post-anesthesia care unit (PACU). They were then transferred to the outpatient unit when they achieved a Modified Aldrete’s score of 9 or higher (range: 0–10; a score of 9 or above indicates that the patient can be discharged from the PACU).^[10]^

### 2.7. Outcome measures

As mentioned earlier, the primary outcome measure of the present study was cardiologist satisfaction level. The cardiologist and patient satisfaction levels were measured on a scale of 1 to 10 (1 = very bad; 10 = very good).

And also, the secondary outcome measures of the present study were incidence of and attempts at successful TEE probe placement, patient satisfaction level, demographic characteristics, procedure data, perioperative and postoperative hemodynamics, and adverse events related to the TEE procedure. The incidence of successful TEE probe placement was expressed as a number, and the attempts at successful TEE probe placement were classified on a scale of first, second, and third. The attempts at successful GLT placement (classified on a scale of first, second, and third), ease of GLT placement (classified on a scale of 1–3: 1 = easy; 2 = moderate; and 3 = hard), and duration of GLT placement (defined as the time from when the anesthesiologist picked up the GLT to when it was successfully placed into the supraglottic area, which was assessed by the presence of meaningful bilateral chest ventilation) were recorded to analyze the performance of the GLT device. Anesthesia duration was defined as the time from which the patient was taken to the procedure room until his/her transfer to the PACU. Procedure duration, defined as the time from the first view to the removal of the TEE probe, was measured and recorded by the cardiologist.

The patients’ hemodynamics (HR, MAP, and SpO_2_) were recorded at various time points: at baseline and at the 1st, 5th, 10th, and 15th min after anesthesia induction. The patients’ demographic characteristics and the procedure data were also recorded.

The adverse events related to the TEE procedure during the perioperative period (dental trauma, cough, bronchospasm, hiccup, hypoxemia, bradycardia, and blood on GLT) and during the postoperative period (headache, nausea and vomiting, and hoarseness and sore throat) were saved by the anesthesiologist. Such adverse events were recorded as 0 or 1 (0 = absent; 1 = present). Hypoxemia is defined as oxygen desaturation indicated by <92% SpO_2_ (determined via pulse oximetry) for longer than 10 s, and bradycardia is defined as an HR of less than 60 beats/min.

### 2.8. Sample size

Statistical power analysis was performed based on a pilot study (5 patients per group) before the investigation of the cardiologist satisfaction levels. Based on these results, we determined that for 80% power and α = 0.05, the measurement of the cardiologist satisfaction levels required 20 patients per group (mean ± standard deviation 8.5 ± 1.4 and 9.5 ± 0.7, respectively). Finally, we calculated that a minimum of 40 patients should be included in the study.

### 2.9. Statistical analysis

The data obtained were analyzed using the Statistical Package for the Social Sciences package program version 22.0 (SPSS Inc., Chicago, IL). The Shapiro–Wilk normality test was used to determine whether the quantitative variables showed a normal distribution. The normally distributed variables in the two groups were compared using the paired-samples *t* test. The abnormally distributed continuous variables were compared using the Mann–Whitney *U* test. The categorical variables were summarized using frequencies and percentages and were compared using the chi-square test or Fisher’s exact test. The descriptive statistics were given as mean, standard deviation, frequency, and percentage (%). The results were evaluated at α 95% confidence interval and at *P* < .05 significance level.

## 3. Results

The two groups’ demographic characteristics (age, gender, height, weight, body mass index, ASA physical status, smoking, and comorbidities) were similar (Table 1).

**Table 1:** Demographic characteristics.

	Group SA (n = 22)		Group GLT (n = 22)		*P* value
	Range	Mean ± std or number (%)	Range	Mean ± std or number (%)	
Age, yr	23–76	52.00 ± 16.93	21–80	49.31 ± 15.63	.588
Gender, n					
Male	–	17 (77.3%)	–	13 (59.1%)	.195
Female	–	5 (22.7%)	–	9 (40.9%)	
Height, cm	155–185	165.40 ± 7.68	155–180	164.59 ± 7.28	.719
Weight, kg	53–100	73.45 ± 12.53	54–96	68.77 ± 11.32	.201
BMI, (kg/m^2^)	20–35	26.78 ± 3.89	20–33	25.39 ± 3.91	.121
ASA physical status, n					
I		2 (9.1%)		0	.291
II		17 (77.3%)		16 (72.7%)	
III		3 (13.6%)		6 (27.3%)	
IV		0		0	
Smoking, n (%)	–	4 (18.2%)	–	2 (9.1%)	.385
Comorbidities, n (%)					
Hypertension	–	6 (%)	–	4 (%)	.060
Thyroid disease	–	2 (%)	–	2 (%)	
Coronary artery disease	–	–	–	3 (%)	
Hypertension + diabetes mellitus	–	2 (%)	–	2 (%)	
Hypertension + coronary artery disease	–	2 (%)	–	1 (%)	
Others	–	5 (%)	–	–	

ASA = American Society of Anesthesiology, BMI = body mass index, cm = centimeter, GLT = gastro-laryngeal tube, SA = sedation and Analgesia, std = standard deviation.

*P* < .05 = statistically significant.

A GLT was successfully placed in 17 patients (77.3%) at the first attempt, in 4 patients (18.2%) at the second attempt, and in 1 patient (4.5%) at the third attempt. GLT placement was easy for 14 patients (63.6%), moderate for 7 patients (31.8%), and hard for 1 patient (4.5%). The mean duration of GLT placement was 21.36 ± 7.96 s.

Both groups had successful TEE probe placements for all the patients. The TEE probe was successfully placed at the first attempt in all the patients in Group GLT and at the first attempt in 11 patients (50.0%), at the second attempt in 8 patients (36.4%), and at the third attempt in 3 patients (13.6%) in Group SA.

The TEE probe placement success in Group GLT was significantly higher than that in Group SA (*P* < .001). TEE probe placement was also significantly easier in Group GLT than in Group SA (*P* < .001). It was easy for 11 patients (50.0%), moderate for 8 patients (36.4%), and hard for 3 patients (13.6%) in Group SA. The propofol consumption (mg), PACU stay duration (min), anesthesia duration (min), and procedure duration (min) were significantly higher in Group GLT than in Group SA (*P* < .001; *P* < .001; *P* = .003; and *P* = .012, respectively). The cardiologist satisfaction level was also significantly higher in Group GLT than in Group SA (*P* = .011), but the two groups’ patient satisfaction levels were similar (*P* = .804). A comparison of the procedure’s characteristics in the two groups is presented in Table 2.

**Table 2:** A comparison of the procedure’s characteristics (GLT or TEE).

	Group SA (n = 22)		Group GLT (n = 22)		*P* value
	Range	Mean ± std or number (%)	Range	Mean ± std or number (%)	
Attempts for successful GLT placement					
First	–	–	–	17 (77.3)	–
Second	–	–	–	4 (18.2)	
Third	–	–	–	1 (4.5)	
Ease of GLT placement					
1 (Easy)	–	–	–	14 (63.6)	–
2 (Moderate)	–	–	–	7 (31.8)	
3 (Hard)	–	–	–	1 (4.5)	
Duration of GLT placement, s	–	–	10–44	21.36 ± 7.96	–
Incidence for successful TEE	–	22	–	22	–
Attempts for successful TEE					
First	–	11 (50.0)	–	22 (100.0)	**<.001** *
Second	–	8 (36.4)	–	–	
Third	–	3 (13.6)	–	–	
Ease of TEE probe placement					
1 (Easy)	–	11 (50.0)	–	22 (100.0)	**<.001** *
2 (Moderate)	–	8 (36.4)	–	–	
3 (Hard)	–	3 (13.6)	–	–	
Propofol consumption, mg	40–200	92.72 ± 39.54	95–280	147.95 ± 54.30	**<.001** *
PACU stay duration, min	2–14	5.59 ± 2.61	7–29	15.31 ± 5.39	**<.001** *
Anesthesia duration, min	7–17	11.54 ± 3.00	10–19	14.27 ± 2.69	**.003** *
Procedure duration, min	6–15	10.04 ± 2.66	8–18	12.13 ± 2.64	**.012** *
Cardiologist satisfaction level	6–10	8.50 ± 1.37	8–10	9.54 ± 0.59	**.011** *
Patient satisfaction level	7–10	9.40 ± 0.79	6–10	9.27 ± 1.03	.804

GLT = gastro-laryngeal tube, PACU = postanesthetic care unit, SA = sedation and analgesia, std = standard deviation, TEE = transesophageal echocardiography.

* *P* < .05 = statistically significant.

There were no significant differences in HR, MAP, and SpO_2_ between the groups at various time points (Table 3). There were also no significant differences between the groups in dental trauma, cough, bronchospasm, hiccup, hypoxemia, bradycardia, and blood on GLT perioperatively and in headache, nausea and vomiting, and hoarseness and sore throat postoperatively. The adverse events related to the TEE procedure are presented in Table 4.

**Table 3:** Heart rate, mean arterial pressure and peripheral oxygen saturation of patients at various time points in two groups.

	Group SA (n = 22)		Group GLT (n = 22)		*P* value
	Range	Mean ± std	Range	Mean ± std	
Heart rate, beat/min					
Baseline	54–123	78.95 ± 16.64	63–118	87.63 ± 13.17	.280
After induction					
1st min	50–106	75.86 ± 13.74	54–107	83.45 ± 12.21	.523
5th min	51–113	72.95 ± 14.88	52–105	79.31 ± 12.48	**.028** *
10th min	58–98	72.06 ± 11.86	55–106	80.54 ± 12.76	.283
15th min	61–63	62.33 ± 1.15	58–105	82.91 ± 11.42	**<.001** *
Mean arterial pressure, mm Hg					
Baseline	75–135	104.68 ± 17.42	82–128	103.18 ± 14.07	.733
After induction					
1st min	50–136	89.09 ± 18.63	70–103	89.18 ± 9.13	.787
5th min	67–117	88.18 ± 14.88	68–115	87.68 ± 11.45	.760
10th min	60–111	86.06 ± 14.13	70–119	88.31 ± 11.42	.631
15th min	61–84	72.00 ± 11.53	83–112	95.58 ± 7.46	.059
Peripheral oxygen saturation, %					
Baseline	96–100	98.63 ± 1.17	97–100	98.22 ± 1.02	.189
After induction					
1st min	95–100	98.72 ± 1.138	97–100	98.68 ± 0.94	.523
5th min	98–100	99.18 ± 0.79	98–100	98.90 ± 0.68	.216
10th min	97–100	98.93 ± 0.88	97–100	98.63 ± 0.84	.283
15th min	98–100	99.33 ± 1.15	98–99	98.66 ± 0.49	.424

GLT = gastro-laryngeal tube, min = minutes, SA = sedation and analgesia, std = standard deviation.

* *P* < .05 = statistically significant.

**Table 4:** Adverse events related to the TEE procedure.

		Group SA (n = 22)	Group GLT (n = 22)	*P* value
		Number (%)	Number (%)	
Dental trauma, n	Perioperative period	–	–	–
Cough, n		2 (9.1)	–	.488
Bronchospasm, n		1 (4.5)	–	1.000
Hiccup, n		1 (4.5)	1 (4.5)	1.000
Hypoxemia, n		–	–	–
Bradycardia, n		–	–	–
Blood on GLT, n		–	5 (22.7)	–
Headache, n	Postoperative period	–	1 (4.5)	1.000
Nausea and vomiting		1 (4.5)	3 (13.6)	.607
Hoarseness, n		–	1 (4.5)	1.000
Sore throat, n		3 (13.6)	5 (22.7)	.698

GLT = gastro-laryngeal tube, SA = sedation and analgesia, TEE = transesophageal echocardiography.

*P* < .05 = statistically significant.

## 4. Discussion

In the present study, Group GLT demonstrated more successful and easier TEE probe placement and a higher cardiologist satisfaction level than Group SA. The TEE probe was placed successfully at the first attempt in nearly 80% of the patients in Group GLT, and was placed easily in nearly 65% of the patients in the same group. As we expected, propofol consumption, PACU stay duration, anesthesia, and TEE procedure duration were significantly higher in Group GLT than in Group SA. The patient satisfaction levels, hemodynamics, and adverse events related to the TEE procedure in the two groups were similar.

TEE is a semi-invasive procedure that utilizes a stiff and flexible endoscope. Thus, cardiologists in many institutions prefer routinely using sedation and analgesia for TEE patients.^[4]^ In this study, we demonstrated GLT use and observed that it provided many benefits, including more successful and easier TEE probe placement, higher cardiologist satisfaction level, elimination of manual airway manipulation during TEE, improved airway management safety, and a detailed TEE view. Thus, we consider GLT a better alternative to sedation and analgesia and to endotracheal intubation for TEE patients. Lateral positioning during TEE with sedation/analgesia needs the application of effective jaw thrust/chin lift maneuvers^[11]^; adequate oxygenation was maintained in Group GLT without requiring maneuvers.

GLT may be inserted into the esophagus by an experienced anesthesiologist with deep sedation and without neuromuscular blockers. The cardiologist must place a mouthpiece into patients’ mouth when using the TEE probe in patients under sedation and analgesia, but there is no need for the cardiologist to wear a mouthpiece when GLT is used. After GLT placement, the TEE probe can easily pass through the GLT’s oropharyngeal canal, and the cardiologist can rapidly obtain cardiac views in the esophagus.

Currently, along with the medical advancement of endoscopic procedures, practitioner and patient satisfaction levels are important markers of the quality of such procedures. The use of airway protective equipment increases practitioner satisfaction.^[12]^ As in previous studies, the present study showed that GLT use significantly increased the cardiologist satisfaction levels (Group SA: 8.50 ± 1.37; Group GLT: 9.54 ± 0.59; *P* = .011). As for the patient satisfaction levels, they were similar in the two groups (Group SA: 9.40 ± 0.79; Group GLT: 9.27 ± 1.03; *P* = .804). We attribute this to the easy and successful GLT placement. The GLT placement was hard in only 1 patient. In addition, the GLT was successfully placed at the third attempt in only 1 patient; it was successfully placed at the first or second attempts in 21 patients.

However, the use of GLT, which is a supraglottic device, causes more sympathetic stimulation and hemodynamic responses, including hypertension and tachycardia, compared to sedation and analgesia. Additionally, airway reflexes must be suppressed via deep anesthesia while using GLT.^[13,14]^ We therefore observed that propofol consumption was significantly higher in Group GLT than in Group SA, as expected. Nonetheless, while a few differences (including in the PACU stay, anesthesia, and procedure durations) between the two groups reached statistical significance, all the results were acceptable, and none of the differences were clinically meaningful. On the other hand, the longer procedure duration in Group GLT is an advantage for cardiologists; they do not need to perform the TEE procedure in a rush, and they can obtain detailed views, as recommended by the American Society of Echocardiography and the Society of Cardiovascular Anesthesiologists.^[15]^

Hemodynamic stability during procedures is important for patients with cardiac problems. The cardiovascular and respiratory depressant effects of anesthetic drugs are the main problems related to patient safety. Thus, anesthesiologists seek to maintain hemodynamic and respiratory stability more optimally during sedation or anesthesia.^[16]^ In this study, hypotensive episodes while using relatively high doses of propofol specifically in Group GLT were the main concern. However, the study results showed that there were no statistical differences in HR and MAP ranges between the two groups, and that such ranges were normal. In addition, we predicted that there would be no perioperative hypoxemia episodes because GLT use improves airway management safety and quality.

Although TEE is considered to have a safety profile, a number of previous studies have reported many kinds of minor and major complications caused by it.^[17]^ Ramalingam et al reported minor complications such as lip/dental/oral injuries, oral bleeding, odynophagia, and swallowing dysfunction and major complications such as upper gastrointestinal bleeding, pharyngeal and esophagogastric tearing, esophageal perforation, and, albeit rarely, death.^[18]^ In the present study, we did not observe any adverse events or complications, such as those reported in the literature. We attributed this to the experienced cardiologist or center in the present study. On the other hand, the incidence of sore throat related to supraglottic airway devices has been described as ranging from 5.8% to 34%.^[19]^ The present study showed that although GLT use more often resulted in sore throat than sedation and analgesia did, this difference between the two groups was not statistically significant. Therefore, GLT can be used without any concern because it does not cause clinically meaningful complications.

Insertion of the TEE probe properly and without causing any damage to it is very important for cost-effectiveness. As the surface of the TEE probe contains piezoelectric crystals, such device is very expensive and has a high risk of being damaged, which can significantly increase the cost of the TEE procedure. Thus, extra care and protection during the probe’s passage through the oral cavity and pharynx are required so that the probe will not incur scratches or tears from the teeth.^[18]^ To prevent such scratches or tears, the patient is generally required to wear a mouthpiece.^[20]^ In the present study, mouthpieces were used for this purpose in Group SA, but there was no need to use a mouthpiece in Group GLT as the GLT’s lumen allows the TEE probe to pass easily and without being damaged through the patient’s mouth, throat, and esophagus.

The use of airway devices in endoscopic procedures is very important to ensure airway management safety. Previous studies have reported that the incidence of hypoxemia in endoscopic procedures, probably caused by a deep sedation level and airway obstruction by the tongue, is very high.^[21,22]^ It may be necessary to terminate the TEE procedure when the utmost airway management safety cannot be achieved.

The present study had some limitations. First, its sample size was relatively small and not adequately powered to assess adverse events related to GLT use or patient satisfaction level. Second, the study was carried out only with ASA I–III patients; therefore, the study results may not apply to ASA IV patients, who have a much higher risk of developing adverse events. Third, we followed up the depth of sedation only clinically, not through bispectral index monitoring. Thus, it is possible that the adverse events that occurred were associated with unmonitored deep sedation levels. Fourth, we did not use capnography, which has been demonstrated to detect depressed respiratory activity before transient hypoxemia.

## 5. Conclusion

TEE, which is gaining increasing popularity for cardiac viewing, may achieve success when optimum anesthetic management is ensured. The present study revealed that GLT use elicited a higher cardiologist satisfaction level and resulted in more successful and easier TEE probe placement. We thus conclude that the use of the recently developed GLT may ensure airway management safety and a comfortable TEE experience.

## Acknowledgments

All procedures performed in studies involving human participants were in accordance with the ethical standards of the institutional and/or national research committee and with the 1964 Helsinki declaration and its later amendments or comparable ethical standards. Informed consent was obtained from the patients for publication of this clinical trial details. This manuscript is edited and revised for clarity, consistency, and correctness according to the requirements and guidelines by Scribendi (ESL Academic Editing Service, https://www.scribendi.com, Order # 879765).

## Author contributions

**Conceptualization:** Muhittin Calim, Harun Uysal, Nuray Kahraman Ay, Kazim Karaaslan, Hayrettin Daskaya.

**Data curation:** Muhittin Calim, Harun Uysal, Nuray Kahraman Ay, Kazim Karaaslan, Hayrettin Daskaya.

**Formal analysis:** Muhittin Calim, Harun Uysal, Nuray Kahraman Ay, Kazim Karaaslan, Hayrettin Daskaya.

**Funding acquisition:** Muhittin Calim, Harun Uysal, Nuray Kahraman Ay, Kazim Karaaslan, Hayrettin Daskaya.

**Investigation:** Muhittin Calim, Harun Uysal, Nuray Kahraman Ay, Kazim Karaaslan, Hayrettin Daskaya.

**Methodology:** Muhittin Calim, Harun Uysal, Nuray Kahraman Ay, Kazim Karaaslan, Hayrettin Daskaya.

**Project administration:** Muhittin Calim, Harun Uysal, Nuray Kahraman Ay, Kazim Karaaslan, Hayrettin Daskaya.

**Resources:** Muhittin Calim, Harun Uysal, Nuray Kahraman Ay, Kazim Karaaslan, Hayrettin Daskaya.

**Software:** Muhittin Calim, Harun Uysal, Nuray Kahraman Ay, Kazim Karaaslan, Hayrettin Daskaya.

**Supervision:** Muhittin Calim, Harun Uysal, Nuray Kahraman Ay, Kazim Karaaslan, Hayrettin Daskaya.

**Validation:** Muhittin Calim, Harun Uysal, Nuray Kahraman Ay, Kazim Karaaslan, Hayrettin Daskaya.

**Visualization:** Muhittin Calim, Harun Uysal, Nuray Kahraman Ay, Kazim Karaaslan, Hayrettin Daskaya.

**Writing – original draft:** Muhittin Calim, Harun Uysal, Nuray Kahraman Ay, Kazim Karaaslan, Hayrettin Daskaya.

**Writing – review & editing:** Muhittin Calim, Harun Uysal, Nuray Kahraman Ay, Kazim Karaaslan, Hayrettin Daskaya.
